# Supramolecular synthon hierarchy in sulfonamide cocrystals with *syn*-amides and *N*-oxides

**DOI:** 10.1107/S2052252519005037

**Published:** 2019-06-21

**Authors:** Geetha Bolla, Ashwini Nangia

**Affiliations:** a School of Chemistry, University of Hyderabad, Gachibowli, Central University P.O., Hyderabad 500 046, India; b Materials Chemistry Division, CSIR-National Chemical Laboratory, Dr. Homi Bhabha Road, Pune 411 008, India

**Keywords:** sulfonamides, *syn*-amides, cocrystals, supramolecular synthons, crystal engineering

## Abstract

Sulfonamide drugs are well known antibacterial and antimicrobial molecules for pharmaceutical development. Building a library of suitable supramolecular synthons for the sulfonamide functional group and understanding their crystal structures with partner coformer molecules continues to be a challenge in crystal engineering. Although a few sulfonamide cocrystals with amides and *N*-oxides have been reported, the body of work on sulfonamide synthons is limited compared with those that have carb­oxy­lic acids and carboxamides. To address this structural gap, the present work is primarily focused on sulfonamide–lactam and sulfonamide–*syn*-amide synthons with drugs such as celecoxib, hydro­chloro­thia­zide and furosemide. Furthermore, the electrostatic potential of previously reported cocrystals has been recalculated to show that the negative electrostatic potential on the lactam and *syn*-amide O atom is higher compared with the charge on carboxamide and pyridine *N*-oxide O atoms. The potential of sulfonamide molecules to form cocrystals with *syn*-amides and lactams are evaluated in terms of the electrostatic potential energy for the designed supramolecular synthons.

## Abstract 2

Crystal engineering of the sulfonamide group with competing coformer molecules (lactam, *syn*-amide and *N*-oxide) using MEP calculations suggests that the SO_2_NH_2_ group will bond to the lactam and *syn*-amide preferentially compared with *N*-oxide and carb­oxy­lic acid, but complexation studies showed superior bonding with *N*-oxide. These results provide a ranking of hydrogen-bonding synthons for crystal engineering with the sulfonamide group.

## 1. Introduction   

Obtaining structural data on supramolecular synthons of the sulfonamide group remains a challenge due to the complexity of this functional group with multiple hydrogen-bond donors and acceptors. Cocrystals of sulfonamides are much less studied compared with carb­oxy­lic acid and carboxamide functional groups even though they have applications for sulfa drugs. A few studies on sulfonamide cocrystals with lactams/*syn*-amides (Bolla *et al.*, 2014 ▸) and pyridine *N*-oxides (Goud *et al.*, 2011 ▸) were reported by some of us. The deliberate assembly of binary and ternary sulfonamide–*syn*-amide cocrystals has been exemplified via benzene­sulfonamide (Bolla *et al.*, 2015 ▸), celecoxib (Bolla *et al.*, 2014 ▸), acetazolamide (Bolla & Nangia, 2016 ▸) and bumetanide (Allu *et al.*, 2017 ▸) drugs, as well as binary and ternary cocrystals with SMBA (*p*-sulfamoyl­benzoic acid; Bolla & Nangia, 2016 ▸), as well as secondary sulfonamide drugs (Elacqua *et al.*, 2013 ▸; Kumar *et al.*, 2017 ▸). These results showed the dominance of the sulfonamide–*syn*-amide supramolecular synthon. For example, Celecoxib–lactam cocrystals crystallized as tri­morphic cocrystals with δ-valerolactam, along with a sul­fon­amide dimer, catemer hydrogen bonds and a carboxamide dimer, whereas the caprolactam cocrystal has a sulfonamide–lactam heterosynthon. The alternation of synthons with even–odd ring coformers provided a systematic analysis of sulfonamide–carboxamide cocrystals (Bolla *et al.*, 2014 ▸). A novel design strategy for binary and ternary cocrystals of the drug acetazolamide (ACZ) (Bolla & Nangia, 2016 ▸) based on the SO_2_NH⋯CONH synthon, together with a size and shape match of coformers (Tothadi *et al.*, 2011 ▸), adds to the background work. This sulfonamide cocrystal approach was illustrated further for the diuretic sulfonamide drug bumetanide (Allu *et al.*, 2017 ▸) with nine binary adducts and four ternary crystalline products. In the present study, the sulfonamide–*syn*-amide synthon is extended to celecoxib (CEL), hydro­chloro­thia­zide (HCT) and furosemide (FUROS) (Scheme 1 *a*). Novel cocrystals with different supramolecular synthons are discussed together with their hydrogen-bonded synthons and molecular electrostatic potential surface energies (MEPSEs). Binary cocrystals of celecoxib with 2HP, MeHP, MeTFHP and OMeHP; hydro­chloro­thia­zide with 2HP, VLM and CPR; and furosemide with 2PY, VLM and CPR are reported (structures of coformers are shown in Scheme 1 *b*) and their single-crystal X-ray structures were analyzed (crystallographic information in Table 1 ▸ and hydrogen-bonding details in Table S1 of the supporting information).

Apart from the detailed structure analysis, the MEPSEs have now been recalculated by DFT 6–311+G** for the library of sulfonamides, *syn*-amides, pyridine carboxamides, carboxamides, carb­oxy­lic acid and pyridine *N*-oxides in different media, such as gas, water, DMF (polar solvent) and THF (non­polar solvent), and their hydrogen-bonding strengths have been ranked. The molecular electrostatic potential energy (MEPE) surfaces and structural data show a competitive hydrogen-bonding hierarchy between the sulfonamide–*syn*-amide and sulfonamide–*N*-oxide supramolecular synthons. Our results show that *syn*-amides are stronger hydrogen-bond acceptors than *N*-oxides based on MEPE-calculated electrostatic charges for predicting competitive hydrogen-bonding preferences in a competitive environment.[Chem scheme1] 
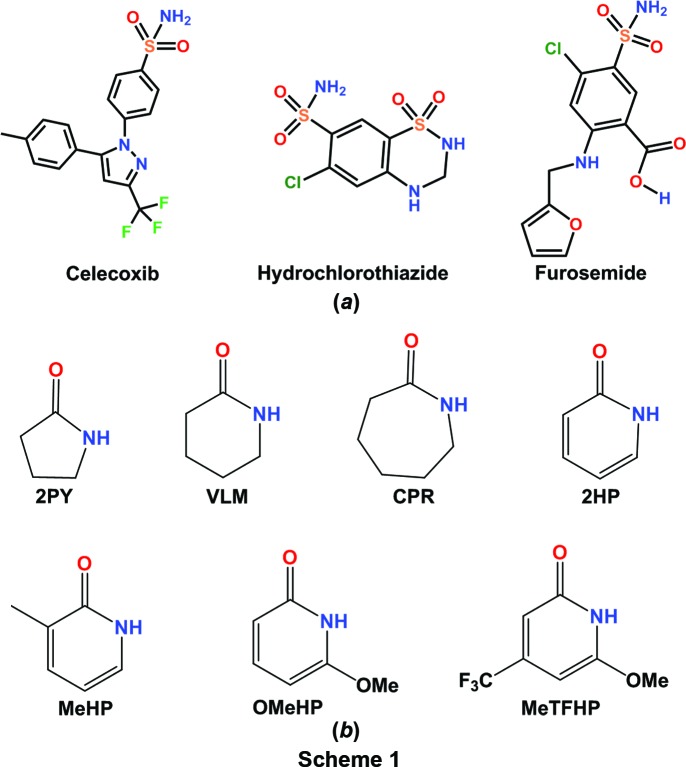


## 2. Experimental   

### 2.1. Preparation of cocrystals   

The sulfonamide drugs CEL, HCT and FUROS (Scheme 1 *a*), and the coformers 2PY, VLM, CPR, 2HP, MeHP, OMeHP and MeTFHP (Scheme 1 *b*) used in this study were purchased from Sigma–Aldrich, Bangalore, India. FUROS and CEL were purchased from Yarrow Chemicals, Mumbai, India. All the solvents used were of analytical grade. Equivalent amounts of the sulfonamide and the appropriate coformer were taken in a pestle and mortar and ground for 20 min using liquid-assisted grinding by adding a few drops of EtOAc. After confirming that the ground mixture is a new solid phase by powder X-ray diffraction (PXRD), the material was dissolved in different solvents (EtOAc:THF and EtOAc:cyclo­hexane) at 50°C until a clear solution appeared. The solution was allowed to reach room temperature and was then filtered by gravity and left aside for slow evaporation. Crystals suitable for X-ray diffraction appeared after 5–6 days.

### 2.2. CEL–2HP (1:1)   

CEL (100 mg, 0.26 mmol) and 2HP (25 mg, 0.26 mmol) were ground for about 20 min by adding 2–3 drops of EtOAc. The ground material was kept for crystallization in EtOAc in a 25 ml conical flask at room temperature. Suitable single crystals were harvested at ambient temperature after one week (m.p. 383 K).

### 2.3. CEL–MeHP (1:1)   

Equimolar quantities of CEL (100 mg, 0.26 mmol) and MeHP (28 mg, 0.26 mmol) were ground for 20 min through liquid-assisted grinding using EtOAc solvent. The ground mixture was dissolved in the optimum amount of EtOAc solvent until the solute dissolved at 40–50°C and then the solution was filtered by gravity and allowed to evaporate at room temperature. Good diffraction-quality single crystals were present after one week (m.p. 388 K).

### 2.4. CEL–MeTFHP (1:1)   

CEL (100 mg, 0.26 mmol) and MeTFHP (46 mg, 0.26 mmol) in a 1:1 ratio were ground for 20 min by liquid-assisted grinding using EtOAc. The ground mixture was dissolved in EtOAc until the solute dissolved at 40–50°C and then the solution was filtered by gravity for crystallization at room temperature. The clear solution afforded good-quality single crystals after one week (m.p. 396 K).

### 2.5. CEL–OMeHP (1:1)   

CEL (100 mg, 0.26 mmol) and OMeHP (33 mg, 0.26 mmol) were ground for 20 min through liquid-assisted grinding using EtOAc. The ground mixture was dissolved in EtOAc until the solute dissolved at 40–50°C and then the clear solution was filtered by gravity to afford diffraction-quality single crystals after one week (m.p. 385 K).

### 2.6. FUROS–2PY-M (2:2:1)   

Equimolar amounts of FUROS (100 mg, 0.30 mmol) and 2PY (28 mg, 0.30 mmol) were ground for 20 min by liquid-assisted grinding using EtOAc. The ground mixture was dissolved in the optimum amount of EtOAc, MeOH, EtOH and THF solvents until the solute dissolved at 40–50°C and the solution was then filtered by gravity. The clear solution was allowed to evaporate at room temperature. Good diffraction-quality single crystals appeared in MeOH as the MeOH solvate after one week (m.p. 388 K). For the other solvents, such as EtOAc, THF, EtOH, CH_3_CN and cyclo­hexane, a precipitate was observed.

### 2.7. FUROS–VLM-H (1:1:1)   

FUROS (100 mg, 0.30 mmol) and VLM (33 mg, 0.30 mmol) were ground for 20 min by liquid-assisted grinding using EtOAc. The ground mixture was dissolved in the optimum amount of EtOAc, MeOH, EtOH and THF solvents until the solute dissolved at 40–50°C. The mixture was filtered by gravity and allowed to evaporate until diffraction quality single crystals appeared in MeOH after one week, confirmed to be the hydrate by single-crystal X-ray data (m.p. 433 K).

### 2.8. FUROS–CPR (1:1)   

Equal amounts of FUROS (100 mg, 0.30 mmol) and CPR (34 mg, 0.30 mmol) were ground for 20 min by liquid-assisted grinding using EtOAc. The ground mixture was dissolved in different solvents, namely EtOAc, MeOH, EtOH and THF, until the solute dissolved at 40–50°C and the solution was then filtered by gravity. The clear solution evaporated to afford good-quality single crystals in MeOH after 3–4 days (m.p. 383 K).

### 2.9. HCT–2HP Form I and Form II (1:1)   

HCT (100 mg, 0.33 mmol) and 2HP (31 mg, 0.33 mmol) were ground for 20 min by liquid-assisted grinding using EtOAc. The ground mixture was dissolved in EtOAc until the solute dissolved at 40–50°C and the solution was left to evaporate at room temperature to yield good diffraction-quality single crystals. Two polymorphs were identified visually: Form I (major) and Form II (minor) appeared concomitantly after 3–4 days. Direct solvent crystallization of HCT and 2HP in a 1:1 ratio often resulted in Form II (m.p. 407 K), whereas grinding the binary mixture for 30 min and recrystallization from EtOAc gave the stable Form I (m.p. 417 K) exclusively.

### 2.10. HCT–VLM (1:2)   

HCT (100 mg, 0.33 mmol) and VLM (33 mg, 0.33 mmol) were ground for 20 min by liquid-assisted grinding using EtOAc. The ground mixture was dissolved in EtOAc until the solute dissolved at 40–50°C and the solution was then filtered by gravity. The clear solution was allowed to evaporate at room temperature. Diffraction-quality single crystals afforded a cocrystal of a 1:2 composition. Pure HCT crystals were also observed in the flask. Continuing the crystallization further gave the bulk cocrystal in a 1:2 stoichiometry (m.p. 398 K).

### 2.11. HCT–CPR (1:2)   

HCT (100 mg, 0.33 mmol) and CPR (36 mg, 0.33 mmol) were ground for 20 min by liquid-assisted grinding using EtOAc. The ground mixture was dissolved in EtOAc at 40–50°C and then the solution was filtered and allowed to evaporate at room temperature. Good diffraction-quality single crystals were obtained in a 1:2 cocrystal stoichiometry along with excess HCT in the flask residue. Further crystallizations continued to yield the 1:2 cocrystal (m.p. 400 K).

### 2.12. Single-crystal X-ray diffraction   

Single crystals were mounted on the goniometer of an Oxford Diffraction Gemini X-ray diffractometer equipped with an Mo *K*α (λ = 0.71073 Å) or Cu *K*α radiation source (λ = 1.54184 Å) at 298 K. Data reduction was performed using *CrysAlis PRO* (Version 1.171.36.28; Agilent Technologies Ltd, 2014; Rigaku Oxford Diffraction Ltd, 2008). The crystal structures were solved and refined using *Olex2* (Dolomanov *et al.*, 2009 ▸), with anisotropic displacement parameters for non-H atoms. H atoms were experimentally located through difference Fourier electron-density maps. In addition, single-crystal X-ray diffraction of the few crystals were collected at 298 K using a Bruker SMART APEX-1 CCD area-detector system equipped with a graphite-monochromated Mo *K*α fine-focus sealed tube (λ = 0.71073 Å) operating at 1500 power, 40 kV and 30 mA. The frames were integrated using *SAINT-Plus* (Bruker, 2003 ▸) with a narrow-frame integration algorithm. The crystal structures were solved and refined using *SHELXT* (Sheldrick, 2015 ▸ *a*) and refined in *SHELXL* (Sheldrick, 2015 ▸ *b*). N—H and O—H hetero-attached H atoms were experimentally located through difference Fourier electron-density maps and carbon-attached H atoms were fixed through using the HFIX instruction. A check of the final CIF using *PLATON* (Spek, 2009 ▸) did not show any missing symmetry. *X-SEED* (Barbour, 2001 ▸) was employed to prepare the figures and packing diagrams. The crystallographic parameters of all the cocrystals are summarized in Table 1 ▸ and hydrogen-bond distances are listed in Table S1 of the supporting information. CIFs are deposited at CCDC Nos. 1860232–1860242.[Chem scheme2] 
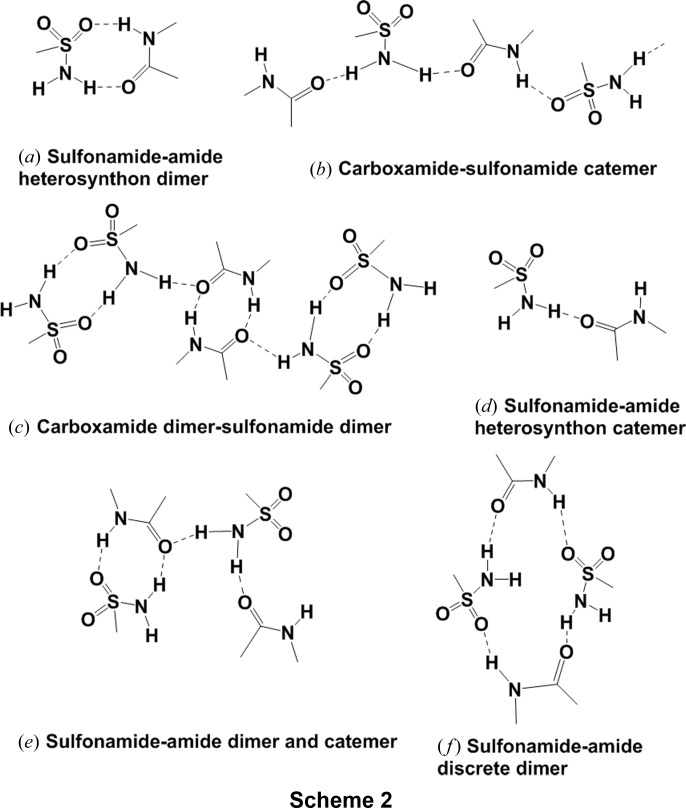


### 2.13. Electrostatic potential calculations   

Molecular electrostatic potential surfaces (MEPS) of the molecules in this study were calculated at the density functional B3LYP level of theory with a 6–311++G** basis set in vacuum, water, non-polar and polar media. All calculations were carried out using *Spartan Student v7* software (Wavefunction Inc., https://www.wavefun.com/). The negative and positive potentials are shown as red and blue surfaces, respectively, indicating the interaction energy value (kJ mol^−1^) of the molecule at that particular atom.

## 3. Results and discussion   

### 3.1. Celecoxib cocrystals   

Celecoxib {4-[5-(4-methyl­phenyl)-3-(triflouro­methyl)-1*H*-pyra­zol-1-yl]benzene­sulfonamide} is a non-steroidal anti-inflammatory drug (NSAID) and specific COX-2 inhibitor for pain and inflammation without inhibiting COX-1. CEL is a Biopharmaceutical Classification System (BCS) Class II drug. The parent drug is labelled as CEL-III (stable polymorph) and a cocrystal of CEL with nicotinamide (CEL–NIC) is reported (Remenar *et al.*, 2007 ▸). These crystal structures were solved by PXRD. We have reported previously cocrystals with lactams (Bolla *et al.*, 2014 ▸) and now we extend our work to sulfonamide synthons (Bolla *et al.*, 2015 ▸) with pyridone cocrystals: CEL–2HP (1:1), CEL–MeHP (1:1), CEL–MeTFHP (1:1) and CEL–OMeHP (1:1). With these additional structural data, we compare the CEL–lactam and CEL–*syn*-amide synthons in sulfonamide structures (Scheme 2). The pyridone cocrystals resulted in supramolecular dimer–catemer and dimer–dimer synthons of sulfonamide with *syn*-amides, similar to CEL-ring lactams (of even number six- or eight-membered-ring lactams), *e.g.* valerolactam (VLM) and aza-2-cyclo­octanone (AZL) (Bolla *et al.*, 2014 ▸).

#### 3.1.1. Crystal structure of CEL–2HP, CEL–MeHP, CEL–OMeHP and CEL–MeTFHP (1:1) cocrystals   

**Figure 1:**
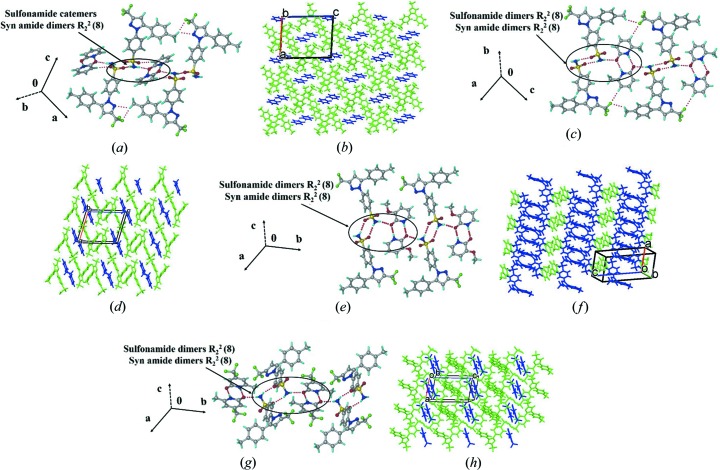
Supramolecular synthons in CEL cocrystals along with hydrogen-bonded synthons and molecular packing.

A single crystal of CEL–2HP (space group *P*2_1_/*n*) is hydrogen bonded through the CEL sulfonamide group with the 2HP dimer in catemer chains [Fig. 1 ▸(*a*)], similar to CEL–VLM Form I crystal packing (Bolla *et al.*, 2014 ▸). There are auxiliary C—H⋯F and C—H⋯O interactions in the structure [Fig. 1 ▸(*b*)]. Among the four CEL cocrystals, CEL–2HP resulted in a dimer–catemer synthon, whereas CEL–MeHP, CEL–OMeHP and CEL–MeTFHP assemble through sulfonamide dimers connected to 2HP dimers [Figs. 1 ▸(*c*), 1(*e*) and 1(*g*)]. The latter synthon matches the reported CEL–VLM Form II crystal structure. The cocrystals of *syn*-amide form dimers because the hydrogen bonding of the CEL sulfonamide group is unable to break the strong coformer hydrogen bonding. The three binary adducts adopt similar 3D crystal packing [Figs. 1 ▸(*d*), 1(*f*) and 1(*h*)] in the same space group (triclinic 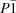).

### 3.2. Hydro­chloro­thia­zide cocrystals   

Hydro­chloro­thia­zide is a diuretic drug which acts by inhibiting the kidneys ability to retain water (Dupont & Dideberg, 1972 ▸) and falls under BCS class IV of low solubility 0.7 g l^−1^ and low permeability log*P* = −0.07 (Amidon *et al.*, 1995 ▸), with bioavailability limited to 65% (Patel *et al.*, 1984 ▸). HCT has four polymorphs, with Forms I (stable phase) and II (less stable phase) reported with 3D coordinates (Kim & Kim, 1984 ▸; Leech *et al.*, 2008 ▸), whereas the other polymorphs are reported by PXRD line profiles. Cocrystals of HCT with piperazine, tetra­methyl­pyrazine, picolinamide, isoniazid, malonamide, nicotinic acid, nicotinamide, succinamide, *p*-amino­benzoic acid, resorcinol, pyrogallol and isonicotinic acid have been reported (Sanphui *et al.*, 2015 ▸; Sanphui & Rajput, 2014 ▸; Gopi *et al.*, 2017 ▸) for improving solubility and membrane permeability. We report a library of synthons for lactam and pyridone derivatives in HCT cocrystals, such as HCT–VLM, HCT–CPR and HCT–2HP polymorphs (Form I and Form II).

#### 3.2.1. Crystal structures of HCT–VLM (1:2) and HCT–CPR (1:2), and polymorphs HCT–2HP (1:1) Form I and HCT–2HP (1:1) Form II cocrystals   

**Figure 2:**
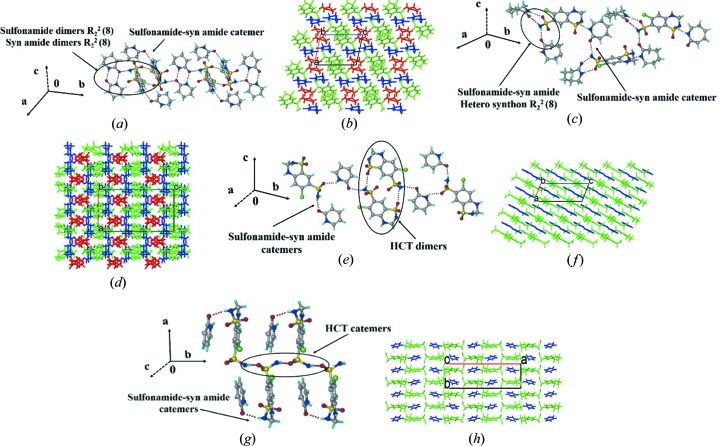
Supramolecular synthons in HCT cocrystals and their molecular packing.

The crystal structure of HCT–VLM (space group 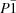) comprises one HCT and two VLM molecules. HCT molecules form homodimers and the primary sulfonamide forms an N—H⋯O heterosynthon with the VLM homodimers and the second VLM forms a catemer chain with the secondary amine of HCT and interacts further with the next neighbour sulfonamide of HCT [Figs. 2 ▸(*a*) and 2(*b*)]. One of the VLM homodimers is sandwiched between the homodimers of HCT and then the second VLM catemer extends with HCT to produce the 2D packing. The crystal structure of HCT–CPR (space group *Pbca*) comprises one HCT and two CPR molecules. Unlike HCT–VLM (1:2), CPR cocrystals contain three different types of heterosynthons [Figs. 2 ▸(*c*) and 2(*d*)]. CPR forms a sulfonamide–lactam heterodimer 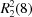 and the *anti* N—H group of SO_2_NH_2_ is connected to the second CPR in the N—H⋯O catemer chain. The amide N—H group of the second CPR forms N—H⋯O interactions with the secondary sulfonamide of HCT such that it acts as a bridge between two HCT molecules. There are no direct HCT dimers as observed in the valerolactam cocrystal. The polymorphs of HCT cocrystals with 2HP (1:1), *i.e.* Form I and Form II, are in the space groups *P*2_1_/*c* and *Pna*2_1_, respectively. The dimers of HCT are connected to the homodimer 2HP, which acts as a bridge between the homodimers of HCT; furthermore, these 1D motifs extend via secondary sulfonamide HCT. In Form II, the primary sulfonamide does not hydrogen bond with the coformer and makes a sulfonamide catemer, whereas the second N—H group forms a heterosynthon with 2HP through an N—H⋯O hydrogen bond. These packing arrangements are displayed in Figs. 2 ▸(*e*), 2(*f*) and 2(*g*), 2(*h*). Form I (m.p. 144°C) and Form II (m.p. 134°C) are monotropically related, as confirmed by differential scanning calorimetry (Fig. S1 of the supporting information).

### 3.3. Furosemide cocrystals   

Furosemide, 4-chloro-2-[(2-furan­ylmethyl)amino]-5-sulfa­moyl­benzoic acid, is a loop diuretic drug used for the treatment of hypertension, hepatic failure and belongs to BCS class IV of low solubility and low permeability. FUROS has two strong hydrogen-bonding functional groups (COOH and SO_2_NH_2_) for crystal engineering. Binary cocrystals of FUROS with acetamide, picolinamide, nicotinamide, isonicotinamide, anthranilamide, tolu­amide, isoniazid, piperazine, tetra­methyl­pyrazine, pyrazine, picolinic acid, *p*-amino­benzoic acid, caffeine, urea, theophylline, adenine, cytosine, bi­pyridines, amino pyridines, pentoxifylline and pyridine *N*-oxides have been reported (Goud *et al.*, 2012 ▸; Harriss *et al.*, 2014 ▸; Sangtani *et al.*, 2015 ▸; Banik *et al.*, 2016 ▸; Stepanovs & Mishnev, 2012 ▸). Five cocrystal polymorphs and one hydrate of FUROS–nicotinamide are reported, and complete crystal structures of FUROS polymorphs I–IV were determined from PXRD data (Ueto *et al.*, 2102 ▸). The structural differences between these polymorphs arise due to changes in the molecular conformation and the hydrogen-bonding synthons. The cocrystals exhibit heterosynthons between the COOH groups of FUROS and the cocrystal polymorphs with nicotinamide are similar to the sulfonamide–amide synthons. The cytosine cocrystal showed synthons, such as the acid–2-aminopyridine salt and sulfonamide–amide, with a *syn*-amide dimer which shows again sulfonamide–lactam and *syn*-amide synthons with furosemide (Fig. S2).

#### 3.3.1. Crystal structure of the FUROS–2PY-M (2:2:1), FUROS–VLM-H (1:1:1) and FUROS–CPR (1:1) cocrystals   

**Figure 3:**
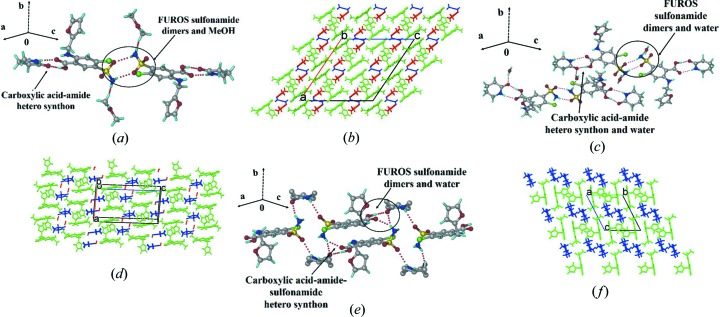
Supramolecular synthons in FUROS cocrystals and their molecular packing.

FUROS–2PY-M crystallized as a methanol solvate in the space group *C*2/*c*. Acid–amide heterodimer 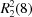 pairs and sulfonamide homodimers are present but a sulfonamide–*syn*-amide synthon is absent in this structure [Fig. 3 ▸(*a*)]. The sulfonamide dimer and acid–amide heterosynthon extend through to MeOH solvate hydrogen bonding [Fig. 3 ▸(**b**)]. The FUROS–VLM-H cocrystal hydrate (space group *P*2_1_/*c*) consists of FUROS and VLM bonded through an acid–amide heterosynthon [Fig. 3 ▸(*c*)] and the sulfonamide homodimers on the other side bond with water producing a 2D structure. The water molecule acts as a bridge for the two adjacent layers similar to the methanol solvate FUROS–2PY-M [Figs. 3 ▸(*b*) and 3(*d*)]. FUROS–CPR crystallizes in the space group 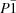 with a sulfonamide–lactam heterosynthon and the C=O group of CPR bonds to the COOH and SO_2_NH_2_ donors [Figs. 3 ▸(*e*) and 3(*f*)].

### 3.4. Molecular electrostatic potential surface energy studies   

**Figure 4:**
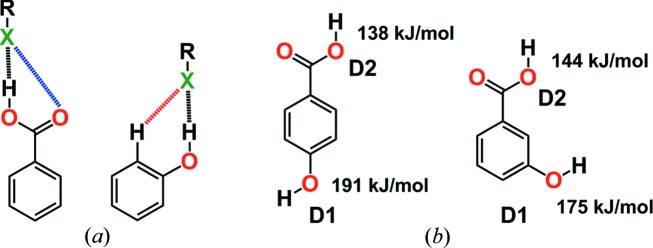
(*a*) Primary and secondary hydrogen-bond interactions. Through-space interactions, the secondary electrostatic interactions (dashed line), make phenol a better hydrogen-bond donor than carb­oxy­lic acid, which has repulsive secondary electrostatic interactions (Hunter, 2004 ▸). (*b*) MEP surface calculations showed that the OH group is the best donor (D1) and the COOH group is the second-best donor (D2) (Aakeröy *et al.*, 2013*a* ▸).

Etter proposed that the best proton donors interact with the best acceptors in the formation of intermolecular hydrogen bonds (Etter *et al.*, 1990 ▸; Etter, 1990 ▸). This ‘rule of thumb’ should be refined for specific functional groups with conformer types to rank the hydrogen-bond donor and acceptor sites matching for crystal engineering. Although this exercise has been successfully demonstrated for functional groups such as COOH, pyridine and CONH_2_ (Aakeröy *et al.*, 2001 ▸, 2005 ▸), data on the sulfonamide group with acceptor atoms in different functional-group environments and in competitive milieu are scarce (publications from our group have been cited in the preceding sections). A general approach was reported (Hunter, 2004 ▸) using calculated molecular electrostatic potential (MEP) energies and molecular design based on the potential interaction free energies of the intermolecular interactions. Based on the calculated MEP surfaces of the hydrogen-bond donor and acceptor sites, it is possible to estimate hydrogen-bond donor–acceptor pairing energies in the solid state, which is a measure of the probability of forming a cocrystal with that supramolecular synthon. The MEP approach was extended for caffeine (Musumeci *et al.*, 2011 ▸) to show that it is sufficiently fast for high-throughput virtual screening and that a balance of MEP and complexation energy must be understood for cocrystal formation. Complementary geometries of 2-methyl­resorcinol, 4,4′-bi­pyridine and planar aromatics, as well as similar shape and size match, are responsible for ternary cocrystal formation (Tothadi *et al.*, 2011 ▸). Aakeröy *et al.* (2013*a* ▸,*b* ▸) and Perera *et al.* (2016 ▸) extended this work to address the importance of MEPE calculations for competing hydrogen-bond and halogen-bond donors. The same authors addressed the question of whether hydrogen-bond interaction ranking is more predictable based on the charge or acidity by selecting a library of ditopic hydrogen-bond donors and acceptors. The phenol OH group is competitive and the preferred hydrogen-bond donor compared with COOH (Aakeröy *et al.*, 2013*a* ▸,*b* ▸; even though COOH is more acidic) when interacting with the pyridine acceptor group [Fig. 4 ▸(*b*)]. These results support Hunter’s explanation (2004) of the through-space effect [Fig. 4 ▸(*a*)], whereby neighbouring atoms in functional groups can perturb the electrostatic potential surface. For example, the carbonyl group of COOH is more electron withdrawing than an aromatic ring, but phenol is a better hydrogen-bond donor than carb­oxy­lic acid (contrary to the acidity rule). The reason is that when a hydrogen-bond acceptor interacts with the phenol O—H group, there is long-range through-space attractive interaction with the adjacent aromatic C—H group, but the corresponding interaction is repulsive for the carbonyl group of COOH. These secondary electrostatic interactions influence the energetics of complexation.

**Figure 5:**
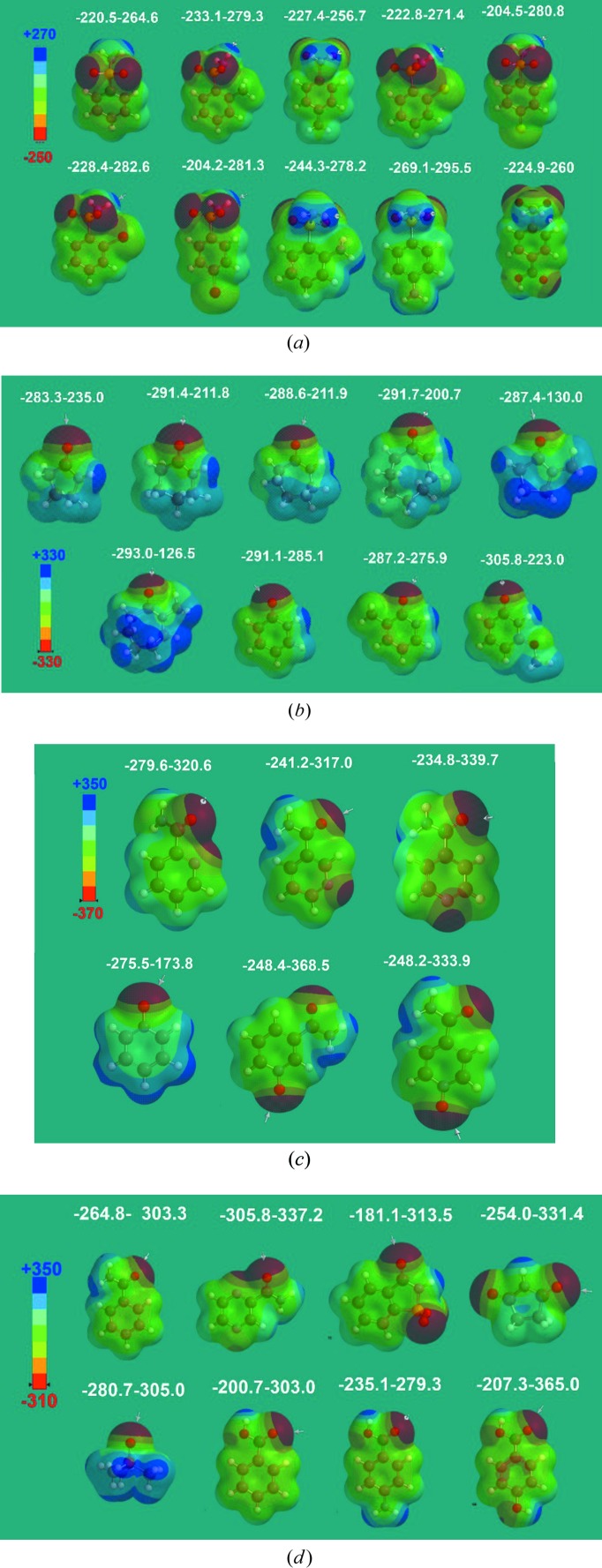
MEPSE (kJ mol^−1^) of the different functional-group molecules. However, for all the coformers (Table S2) used in the present study, MEPSEs are calculated in different media such as gas, water, polar (DMF) and non-polar (THF) solvents (Table S3).

Recently, Kent *et al.* (2018 ▸) calculated the electrostatic potential maps for the high-nitro­gen energetic material 3,6-bis­(1*H*-1,2,3,4-tetrazol-5-yl­amino)-s-tetrazine (BTATz) with a library of coformers. They showed that the C=O acceptor (of 2-pyridone) is more electronegative (−223.2 kJ mol^−1^) compared with the *N*-oxide (−204.6 kJ mol^−1^) by the density functional method B3LYP/6–31+G** in the gas phase. In this background, MEPE calculations on *syn*-amide and *N*-oxide, the two acceptor groups for the SO_2_NH_2_ donor group in our previous studies (Bolla *et al.*, 2015 ▸; Bolla & Nangia, 2015 ▸, 2016 ▸; Allu *et al.*, 2017 ▸; Goud *et al.*, 2011 ▸) were performed to show that the amide C=O group is more electronegative compared with *N*-oxide coformers. MEP energies were calculated using *Spartan* for primary sulfonamide cocrystals with lactam, *syn*-amide, pyridine carboxamide, carboxamide, *N*-oxide and carb­oxy­lic acid groups in gas and aqueous media (Figs. 5 ▸ and 6, ▸ and Fig. S3), and polar (DMF) and nonpolar (THF) solvents (see Table S2 for all molecular structures and Table S3 for all calculated energy values in different media). The negative electrostatic potentials of lactam and *syn*-amide, *i.e.* −280 to −305 kJ mol^−1^ (in water, other values are listed in Table S3), are more negative than *N*-oxide at −248 to −275 kJ mol^−1^ and the sulfonamide group at −220 to −230 kJ mol^−1^. These values mean that the lactam or *syn*-amide C=O group is a better hydrogen-bond acceptor when compared with *N*-oxide for the sulfonamide N—H donor. The *N*-oxide of nicotinamide and isonicotinamide are strong acceptors. MEPSE calculations confirm that the C=O groups of primary carboxamides and carb­oxy­lic acids are weaker accepters than lactam and *N*-oxide, as expected from functional-group chemistry. The negative MEPSE of the sulfonamide SO_2_ group indicates a weak acceptor and, similarly, the positive MEPSE of the N—H groups of lactams/*syn*-amides are weak donors, which is consistent with the target cocrystals formed and observed. Calculations in different media showed similar results (Table S3). The reported sulfonamide–amide and sulfonamide–*N*-oxides competitive studies are analyzed computationally in this article. Further experiments on slurry grinding and solvent-assisted grinding are pending. This is the first such article from our group (and on this subject with respect to sulfonamides in the published literature) suggesting that more experiments are required to fully understand this pharmaceutically interesting system.

### 3.5. Complexation energy studies   

**Figure 7:**
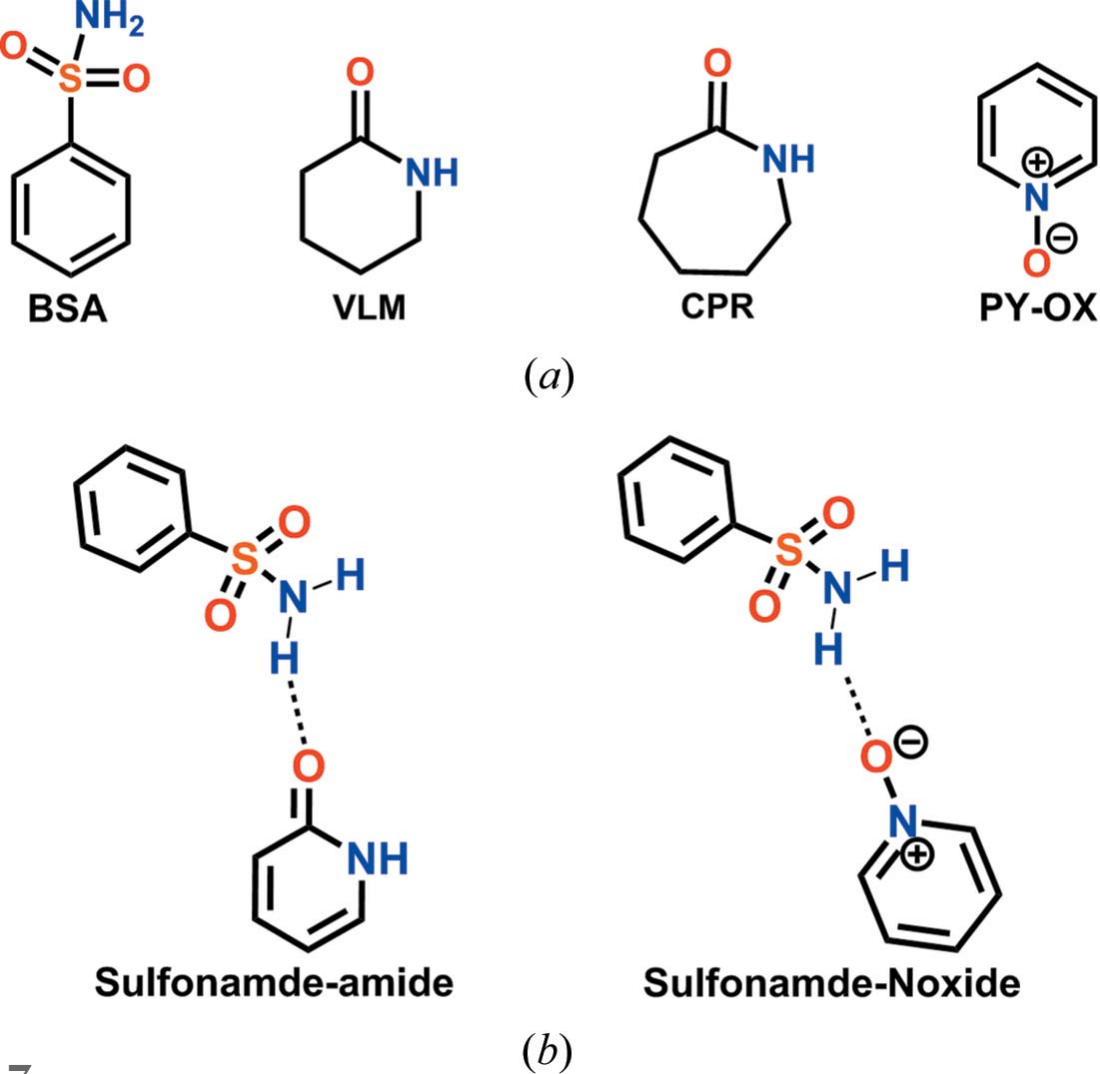
(*a*) The molecular structures of benzenesulfonamide, lactam and *N*-oxide. (*b*) Supramolecular synthons with *N*-oxide and *syn*-amide motifs.

The structures with the minimum stabilization energy of the hydrogen-bonded complex were calculated using *Materials Studio* in the Dreiding force field (http://accelrys.com/products/collaborative-science/biovia-materials-studio/). The complexation energy was calculated as the difference between the optimized complex and the combined energy of the optimized individual molecules. Thus, *E*_AB_ (A = sulfonamide, B = coformer and AB = cocrystal) is the energy of the optimized molecular complex [see Equation (1)], and *E*_A_ and *E*_B_ are the energies of the optimized starting materials, sulfonamide and coformer, *e.g.* lactam and *N*-oxide [see Equation (1) and Fig. 7 ▸].
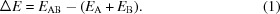
Benzenesulfonamide with valerolactam and caprolactam cocrystals were studied and the work suggested that the Δ*E* values of both cocrystals were close (BSA–VLM: −10.06 kcal mol^−1^; BSA–CPR: −11.23 kcal mol^−1^). Furthermore, benzenesulfonamide with pyridine *N*-oxide (Table 2 ▸) was calculated to be −16.32 kcal mol^−1^. Thus, the complexation energies of the lactam and *syn*-amides are very close and stronger than that of lactone. However, *N*-oxide gives a more stable complex (Table 2 ▸).

## 4. Conclusions   

Cocrystals of sulfonamide drugs, such as celecoxib, hydro­chloro­thia­zide and furosemide, are reported with lactams and *syn*-amides. To better understand the concept of the sulfonamide donor with multiple acceptor coformers, the energy and enthalpic advantage in heterosynthons and cocrystal formation MEPSES were calculated in different media, such as gas, water, nonpolar (THF) and polar (DMF) solvents. There is a competition and interplay of the interactions and energies with lactam and *syn*-amide to form reproducible synthons in cocrystals. The molecular electrostatic potential surface of sulfonamide cocrystals with the acceptor-group coformers suggest strong hydrogen bonding with lactam and *syn*-amide when compared with *N*-oxide, carboxamide and carb­oxy­lic acid. These results not only rationalize the formation of the previously reported sulfonamide cocrystals, but more importantly present a hierarchy for planning future studies on cocrystals of the sulfonamide drugs category.

Crystal engineering of the sulfonamide group with com­peting coformer molecules (lactam, *syn*-amide and *N*-oxide) using MEP calculations suggest that the SO_2_NH_2_ group will bond with lactam and *syn*-amide preferentially compared with *N*-oxide and carb­oxy­lic acid, but complexation studies showed superior bonding with *N*-oxide. These results provide a ranking of hydrogen-bonding synthons for crystal engineering with the sulfonamide group.

## Supplementary Material

Crystal structure: contains datablock(s) CEL2HP, CELMeHP, CELMeTFHP, CELOMeHP, FUROS2PY, FUROSCPR, FUROSVLM, HCT2HPFORMI, HCTHPFORMII, HCTVLM12, HCTCPR12. DOI: 10.1107/S2052252519005037/lq5020sup1.cif

Structure factors: contains datablock(s) CEL2HP. DOI: 10.1107/S2052252519005037/lq5020CEL2HPsup2.hkl

Structure factors: contains datablock(s) CELMeHP. DOI: 10.1107/S2052252519005037/lq5020CELMeHPsup3.hkl

Structure factors: contains datablock(s) CELMeTFHP. DOI: 10.1107/S2052252519005037/lq5020CELMeTFHPsup4.hkl

Structure factors: contains datablock(s) CELOMeHP. DOI: 10.1107/S2052252519005037/lq5020CELOMeHPsup5.hkl

Structure factors: contains datablock(s) FUROS2PY. DOI: 10.1107/S2052252519005037/lq5020FUROS2PYsup6.hkl

Structure factors: contains datablock(s) FUROSCPR. DOI: 10.1107/S2052252519005037/lq5020FUROSCPRsup7.hkl

Structure factors: contains datablock(s) FUROSVLM. DOI: 10.1107/S2052252519005037/lq5020FUROSVLMsup8.hkl

Structure factors: contains datablock(s) HCT2HPFORMI. DOI: 10.1107/S2052252519005037/lq5020HCT2HPFORMIsup9.hkl

Structure factors: contains datablock(s) HCTHPFORMII. DOI: 10.1107/S2052252519005037/lq5020HCTHPFORMIIsup10.hkl

Structure factors: contains datablock(s) HCTVLM12. DOI: 10.1107/S2052252519005037/lq5020HCTVLM12sup11.hkl

Structure factors: contains datablock(s) HCTCPR12. DOI: 10.1107/S2052252519005037/lq5020HCTCPR12sup12.hkl

Supporting Information. DOI: 10.1107/S2052252519005037/lq5020sup13.pdf

CCDC references: 1860232, 1860233, 1860234, 1860235, 1860236, 1860237, 1860238, 1860239, 1860240, 1860241, 1860242

## Figures and Tables

**Figure 6:**
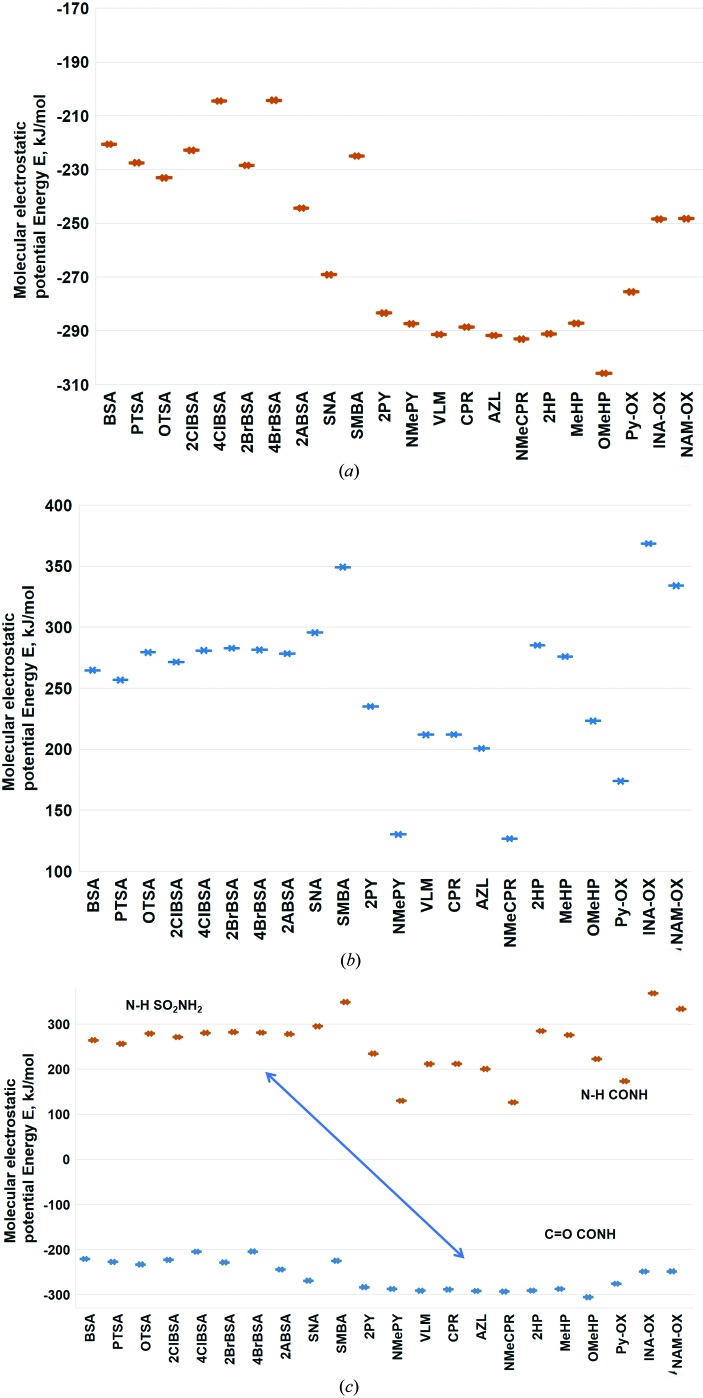
(*a*) Negative electrostatic potentials (in kJ mol^−1^) show that lactam and *syn*-amide are more electronegative that *N*-oxide coformers. (*b*) Lactam and *syn*-amide are less electropositive (in kJ mol^−1^) compared with other coformers. (*c*) Comparison of the positive and negative electrostatic potential energy. All the structures and energy values are displayed in Fig. S3 and Tables S2 and S3.

**Table:** 

	CEL–2HP (1:1)	CEL–MeHP (1:1)	CEL–MeTFHP (1:1)	CEL–OMeHP (1:1)	FUROS–2PY-M (2:2:1)
CCDC code	1860232	1860233	1860234	1860235	1860236
Chemical formula	C_17_H_14_F_3_N_3_O_2_S·C_5_H_5_NO	C_17_H_14_F_3_N_3_O_2_S·C_6_H_7_NO	C_17_H_14_F_3_N_3_O_2_S·C_7_H_6_F_3_NO	C_17_H_14_F_3_N_3_O_2_S·C_6_H_7_NO_2_	2(C_12_H_11_ClN_2_O_5_S)·2(C_4_H_7_NO)·C_2_H_7_O
Formula weight	476.47	490.50	558.50	506.50	878.76
Crystal system, space group	Monoclinic, *P*2_1_/*n*	Triclinic, 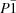	Triclinic, 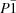	Triclinic, 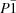	Monoclinic, *C*2/*c*
Temperature (K)	298	298	298	298	298
*a* (Å)	14.5182 (15)	10.0694 (8)	7.4563 (9)	7.6112 (9)	23.819 (3)
*b* (Å)	8.2844 (12)	10.6113 (10)	13.0100 (15)	11.3462 (14)	8.4372 (5)
*c* (Å)	17.8349 (18)	12.6499 (14)	13.7231 (16)	15.1063 (18)	23.450 (2)
α (°)	90	113.451 (10)	100.170 (2)	105.897 (2)	90
β (°)	93.899 (9)	100.897 (8)	95.715 (2)	102.702 (2)	123.868 (16)
γ (°)	90	101.744 (7)	104.444 (2)	101.930 (2)	90
*V* (Å^3^)	2140.1 (4)	1157.9 (2)	1254.4 (3)	1173.4 (2)	3913.0 (9)
*D*_calc_ (g cm^−3^)	1.479	1.407	1.479	1.434	1.492
*Z*	4	2	2	2	4
μ (mm^−1^)	0.21	0.20	0.21	0.20	0.35
No. of measured, independent, observed [*I* > 2σ(*I*)] reflections	12941, 3634, 2269	8277, 4717, 3080	12175, 4420, 3763	12619, 4786, 4107	7270, 3327, 2676
*R*_int_	0.067	0.026	0.031	0.029	0.024
*R*[*F* ^2^ > 2σ(*F* ^2^)], *wR*(*F* ^2^)	0.049, 0.108	0.068, 0.205	0.053, 0.138	0.061, 0.174	0.060, 0.172
Goodness-of-fit	0.99	1.03	1.08	1.03	1.06
Diffractometer, radiation type	Xcalibur, Eos, Gemini, Mo *K*α	Xcalibur, Eos, Gemini, Mo *K*α	CCD area detector, Mo *K*α, Bruker SMART APEX-I	CCD area detector, Mo *K*α, Bruker SMART APEX-I	Xcalibur, Eos, Gemini, Mo *K*α

**Table:** 

	FUROS–VLM-H (1:1:1)	FUROS–CPR (1:1)	HCT–2HP, FORM I (1:1)	HCT–2HP, FORM II (1:1)	HCT–VLM (1:2)	HCT–CPR (1:2)
CCDC code	1860238	1860237	1860239	1860240	1860241	1860242
Chemical formula	C_12_H_11_ClN_2_O_5_S·C_5_H_9_NO·H_2_O	C_12_H_11_ClN_2_O_5_S·C_6_H_11_NO	C_7_H_8_ClN_3_O_4_S_2_·C_5_H_5_NO	C_7_H_8_ClN_3_O_4_S_2_·C_5_H_5_NO	C_7_H_8_ClN_3_O_4_S_2_·2(C_5_H_9_NO)	C_7_H_8_ClN_3_O_4_S_2_·2(C_6_H_11_NO)
Formula weight	447.88	443.89	392.83	392.83	496.00	524.05
Crystal system, space group	Monoclinic, *P*2_1_/*n*	Triclinic, 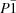	Monoclinic, *P*2_1_/*c*	Orthorhombic, *Pna*2_1_	Triclinic, 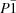	Orthorhombic, *Pbca*
Temperature (K)	298	298	298	298	100	298
*a* (Å)	11.116 (5)	8.5442 (6)	6.8039 (5)	29.442 (4),	8.6930 (6)	11.8873 (12)
*b* (Å)	8.447 (2)	11.3615 (8)	13.5399 (8)	7.3421 (9),	10.6472 (7)	19.315 (2)
*c* (Å)	21.388 (7)	12.1409 (8)	18.8949 (13)	7.0867 (7)	12.8556 (9)	21.733 (2)
α (°)	90	63.447 (1)	90	90	113.672 (1)	90
β (°)	93.12 (3)	88.724 (1)	113.113 (9)	90	A1	z90
γ (°)	90	75.712 (1)	90	90	95.358 (1)	90
*V* (Å^3^)	20050.4 (12)	1016.37 (12)	1601.0 (2)	1531.9 (3)	1060.61 (13)	4990.0 (9)
*D*_calc_ (g cm^−3^)	1.483	1.450	1.630	1.703	1.553	1.395
*Z*	4	2	4	4	2	8
μ (mm^−1^)	0.34	0.33	4.87	0.56	0.42	0.36
No. of measured, independent, observed [*I* > 2σ(*I*)] reflections	7859, 3402, 1676	9612, 3456, 3040	5295, 2853, 2452	3787, 2439, 1499	11318, 4289, 4106	49128, 4922, 4329
*R*_int_	0.074	0.024	0.024	0.056	0.024	0.028
*R*[*F* ^2^ > 2σ(*F* ^2^)], *wR*(*F* ^2^)	0.065, 0.114	0.054, 0.157	0.072, 0.199	0.081, 0.128	0.031, 0.082	0.036, 0.102
Goodness-of-fit	0.99	1.07	1.16	1.09	1.06	1.03
Diffractometer, radiation type	Xcalibur, Eos, Gemini, Mo *K*α	CCD area detector, Mo *K*α, Bruker SMART APEX-I	Xcalibur, Eos, Gemini, Cu *K*α	Xcalibur, Eos, Gemini, Mo *K*α	CCD area detector, Mo *K*α, Bruker SMART APEX-I	CCD area detector, Mo *K*α, Bruker SMART APEX-I

**Table 2:** Geometry-optimized energy of the starting material, complexes and their difference (kcal mol^−1^)

Compound	*E*_A_	*E*_B_	*E*_AB_	Δ*E* = *E*_AB_ − (*E*_A_ + *E*_B_)
BSA–VLM	−48.178094	−32.366595	−90.608624	−10.06393
BSA–CPR	−48.178094	−30.987992	−90.401877	−11.235791
BSA–PY-OX	−48.178094	−49.388313	−113.887402	−16.320995
