# Supplementary material for: Supramolecular synthon hierarchy in sulfonamide cocrystals with syn-amides and N-oxides
Source: IUCrJ. 2019 Jun 21;6(Pt 4):751–60. doi: 10.1107/S2052252519005037 (PMC6608642; doi:10.1107/S2052252519005037)
Supplement: Supplementary file 13 [file m-06-00751-sup13.pdf]

IUCrJ

Volume 6 (2019)

Supporting information for article:

Supramolecular synthon hierarchy in sulfonamide cocrystals with *syn*-amides and *N*-oxides

Geetha Bolla and Ashwini Nangia

X-ray powder diffraction

Bulk samples were analyzed by X-ray powder diffraction on a Bruker AXS D8 diffractometer (Bruker-AXS, Karlsruhe, Germany). Experimental conditions: Cu K radiation (λ = 1.54056 Å), 40 kV, 30 mA, scanning interval 5–50 2θ.

Thermal analysis Differential Scanning Calorimetry (DSC)

DSC was performed on a Mettler Toledo DSC 822e module. Samples were placed in crimped but vented aluminium sample pans, with a typical sample weighed of 3–5 mg. The temperature range was set 30–200 ˚C min^{-1} at a heating rate of 10 ˚C min. While sample running it was purged with a stream of dry N_2 flow at 80 ml min^{-1}.

Table S1 Hydrogen bond geometry of the crystal structures in this present study.

Crystal	Interaction	H···A /Å	D···A /Å	∠D–H···A /º	Symmetry code
CEL–2HP (1:1)	C16–H16···N1	3.01	3.376(4)	105	1-X,-Y,-Z
	C10–H10···F3	2.59	3.321(4)	136	1-X,1-Y,-Z
	N3–H3A···O3	2.06(3)	2.925(4)	171	1/2+X,1/2-Y,-1/2+Z
	N3–H3B···O2	2.22(3)	3.072(4)	166	3/2-X,1/2+Y,-1/2-Z
	C11–H11A··· N1	2.79	3.464(4)	127	1/2+X,1/2-Y,1/2+Z
	N4–H4···F1	2.80	3.092(3)	101	1/2+X,1/2-Y,1/2+Z
	N4–H4···O3	1.94	2.800(4)	178	1-X,-Y,1-Z
	C19–H19···O1	2.59	3.492(5)	162	-1/2+X,1/2-Y,1/2+Z
CEL–MeHP	N4–H4A···O2	2.18	2.994(3)	157	1-X,2-Y,1-Z
	N1–H1···O1	1.97	2.823(3)	178	-X,2-Y,-Z
	N4–H4B···O1	2.09	2.882(3)	173	-X,2-Y,-Z
CEL–OMeHP (1:1)	C16–H16···O2	2.53	2.897(3)	104	1-X,2-Y,1-Z
	N3–H3A···O1	2.23	2.987(3)	167	-X,-Y,-Z
	N3–H3B···O3	2.07	2.893(3)	161	1+X,-1+Y,+Z
	N4–H4···O3	1.91	2.800(3)	175	-X,1-Y,-Z
CEL–MeTFHP (1:1)	N1–H1···O1	1.97	2.823(3)	178	1-X,1-Y,-Z
	N4–H4B···O1	2.09	2.882(3)	173	1/2+X,1/2-Y,-1/2+Z
	N4–H4A···O2	2.18	2.994(3)	157	1-X,-Y,-Z
HCT–VLM (1:2)	C16–H16B··· O1	2.75	3.2364(18)	114	+X,+Y,1+Z
	C15–H15B··· O4	2.65	3.3388(19)	126	2-X,1-Y,1-Z
	N5–H5··· O4	2.36	3.1681(16)	159	+X,1+Y,1+Z
	N4–H4A···O5	2.08	2.9055(17)	177	1-X,1-Y,-Z
	N2–H2···O3	2.21	2.9148(16)	142	2-X,1-Y,1-Z

	N1–H1···O6	2.03	2.8357(17)	172	2-X,1-Y,1-Z
	N3–H3A···O6	2.17	2.9508(17)	154.4	1-X,1-Y,1-Z
HCT–CPR (1:2)	C4–H4···O5	2.55	3.303(2)	137	1/2+X,1/2-Y,1-Z
	C9–H9B···O1	2.84	3.554(2)	131	1-X,1/2+Y,3/2-Z
	N4–H4A···O3	2.21	2.9831(19)	169	-1+X,+Y,+Z
	N1–H1···O5	2.20	2.943(2)	154	1/2+X,1/2-Y,1-Z
	N5–H5···O1	2.21	3.024(2)	176	1-X,-Y,1-Z
	N2–H2···O6	2.00	2.811(2)	154	1+X,+Y,+Z
	N3–H3A···O6	2.08	2.917(2)	171	1-X,-Y,1-Z
HCT–2HP Form I (1:1)	C4–H4···O5	2.55	3.303(2)	137	1+X,3/2-Y,1/2+Z
	C7–H7A···O3	2.68	3.025(2)	101	1-X,1/2+Y,1/2-Z
	C13–H13A···O2	2.52	3.483(2)	175	2-X,-1/2+Y,1/2-Z
	C18–H18A···O5	3.00	3.473(3)	111	2-X,2-Y,-Z
	N4–H4A···O3	2.21	2.9831(19)	169	2-X,1-Y,1-Z
	N1–H1···O5	2.20	2.943(2)	154	1+X,3/2-Y,1/2+Z
	N1–H1···O4	2.88	3.2023(19)	106	1-X,1/2+Y,1/2-Z
	N5–H5···O1	2.21	3.024(2)	176	+X,-1+Y,1+Z
	N2–H2···O6	2.00	2.811(2)	154	-1+X,3/2-Y,1/2+Z
	N3–H3A···O6	2.08	2.917(2)	171	+X,-1+Y,1+Z
	N3–H3B···O5	2.05	2.847(2)	167	+X,3/2-Y,1/2+Z
	C4–H4···O1	2.455	3.358(10)	163	1+X, 3/2-Y, 1/2+Z
HCT–2HP Form II (1:1)	N2–H2···O2	3.10	3.463(9)	111	+X,+Y,-1+Z
	N3–H3A···O4	2.23	2.971(8)	166	2-X,1-Y,-1/2+Z
	N3–H3B···O3	1.88	2.849(8)	168	2-X, 2-Y,-1/2+Z
	C9–H9···O1	2.661	3.326(11)	129	3/2X,1/2+Y,1/2+Z
	C11–H11···O3	2.665	3.560(13)	161	3/2-X,1/2+Y,-1/2+Z
	N4–H4A···N2	2.6	3.174(14)	127	+X,-1+Y,+Z
FUROS–2PYM (2:2:1)	N1–H1A···O6	2.43	3.437(6)	150	+X,-Y,- 1/2+Z
	N1–H1B···O1	2.82	3.000(5)	137	1/2-X,0.5-Y, Z
	N3–H3A···O3	2.12	2.929(4)	155	-X,+Y,3/2-Z
	C12–H12···O1	2.59	3.483(6)	160	1/2-X, 1/2+Y, 1/2-Z
	C17–H17B···N2	2.44	3.356(6)	159	-X,1-Y,1-Z
	N1–H1A···O6	2.82	3.437(6)	150	+X,-Y,- 1/2+Z
	N1–H1B···O1	2.43	3.000(5)	137	1/2-X, 1/2-Y,-Z
FUROS–CPR (1:1)	N3–H3···O1	2.28	3.016(3)	143	1-X,2-Y,2-Z
	C7–H7···O2	2.42	2.839(3)	107	1-X,1-Y,1-Z
	C8–H8A···O4	2.63	3.359(3)	132	1+X,+Y,+Z

	C10–H10···O2	2.52	3.274(5)	138	1+X,+Y,+Z
	C12–H12···O1	2.64	3.398(5)	139	2-X,1-Y,2-Z
	N1–H1A···O3	2.17	2.966(4)	152	1-X,2-Y,2-Z
	N1–H1A···O6	2.54	3.067(4)	120	1-X,2-Y,1-Z
	N2–H2···O3	2.03	2.660(3)	143	1-X,2-Y,2-Z
	N1–H1B···O1	2.44	3.163(4)	157	1-X,2-Y,1-Z
	O4–H4···O6	1.55	2.575(3)	174	+X,+Y,1+Z
FUROS–VLMH (1:1:1)	N2–H2···O3	2.04	2.673(4)	129	3/2-X,1/2+Y,1/2-Z
	N2–H2··· O7	2.79	3.379(6)	127	+X,1+Y,+Z
	N1–H1A···O1	2.22	3.057(5)	161	2-X,1-Y,-Z
	N1–H1B··· O7	2.03	2.940(6)	171	3/2-X,1/2+Y,1/2-Z
	C8–H8A···O7	2.94	2.675(6)	102	+X,1+Y,+Z
	C8–H8A··· O4	2.54	2.812(5)	108	+X,1+Y,+Z
	C17– H17B···N1	2.81	2.977(5)	164	3/2-X,1/2+Y,1/2-Z
	C10– H10···O2	2.75	3.288(5)	133	+X,1+Y,+Z
	C12– H12···O1	2.39	3.494(6)	117	5/2-X,1/2+Y,1/2-Z
	O7– H7B···O5	2.67	3.459(6)	103	+X,-1+Y,+Z
	O7– H7B···O7	2.84	3.494(7)	128	2-X,-Y,1-Z

Table S2 Molecular structures of the all the components in this study.

Sulfonamide's				
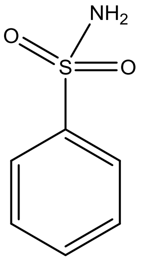 BSA	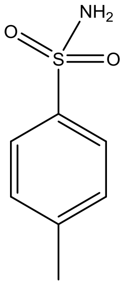	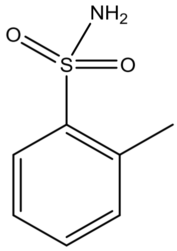 OTSA	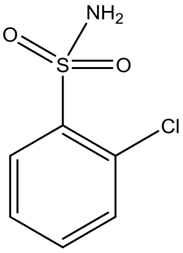 2ClBSA	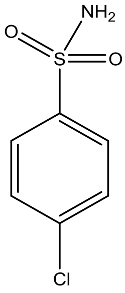

	PTSA			4ClBSA
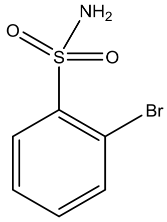 2BrBSA	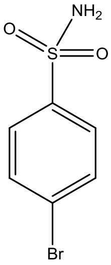 4BrBSA	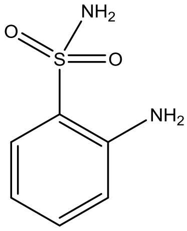 2ABSA	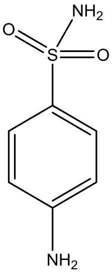 SNA	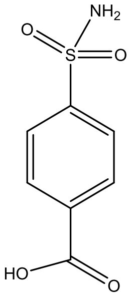 SMBA
Lactams, syn amides				
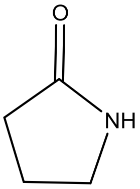 2PY	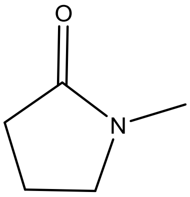 NMe2PY	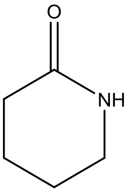 VLM	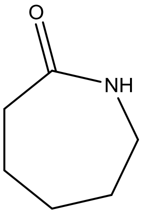 CPR	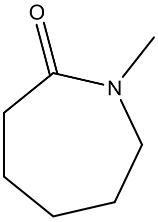 NMeCPR
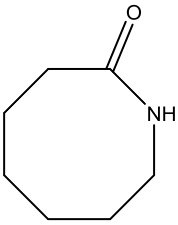 AZL	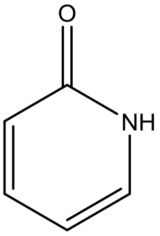 2HP	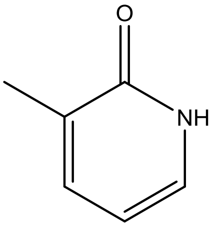 MeHP	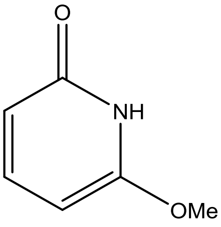 OMeHP	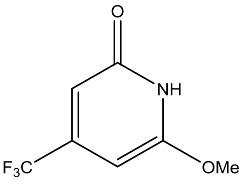 MeTFHP
N-oxides				
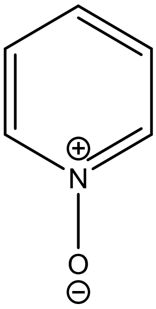 PY-OX	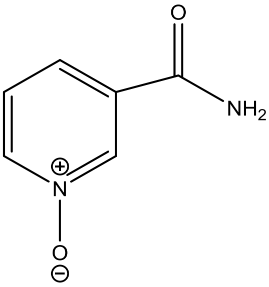 NAM-OX	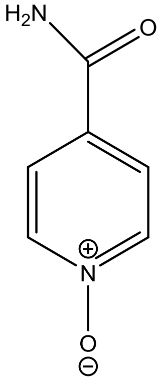 INA-OX		
Pyridine carboxamides				

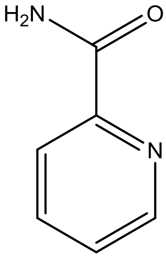

PAM

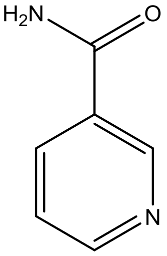

NAM

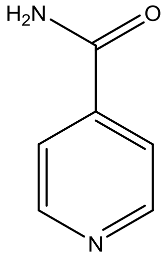

INA

Amides

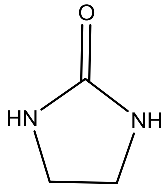

MAM

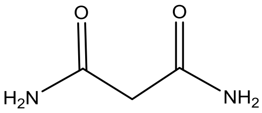

SCA

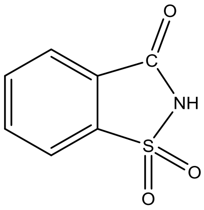

SAC

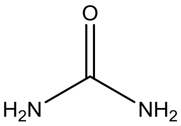

URA

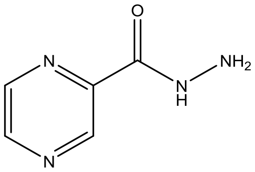

INZ

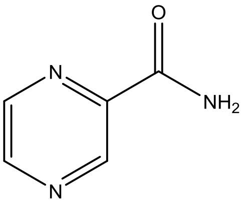

PZA

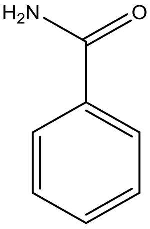

BZA

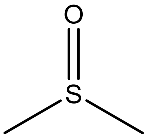

DMSO

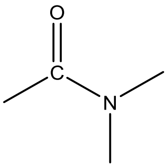

DMF

Acid coformers

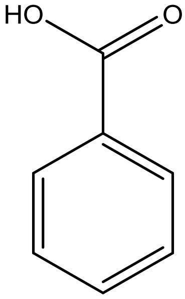

BA

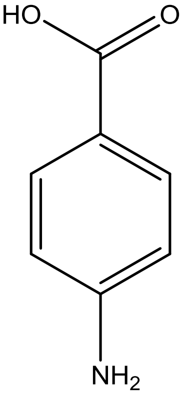

PABA

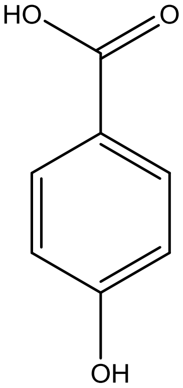

POHBA

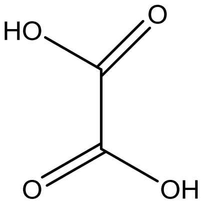

OA

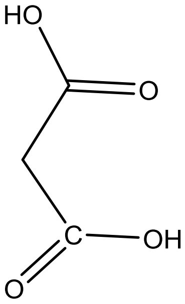

MA

Table S3 Molecular electrostatic potentials energy calculations of sulphonamides and coformers using Spartan Student v7 software, Wavefunction, Inc., https://www.wavefun.com/ dated 27-01-2018. The calculated MEPE values are listed.

Molecule	DFT, 6-311+G**			
	E, kJ/mol (gas)	E, kJ/mol (water)	E, kJ/mol (polar) (DMF)	E, kJ/mol (non-polar) (THF)

Sulfonamide				
BSA	-174.36, -216.11	-220.59, -264.68	-219.87, -264.02	-212.57, -256.63
PTSA	-178.87, -210.68	-227.479, -256.699	-226.729-, 256.041	-219.305, -249.151
OTSA	-177.055, -217.295	-233.11, -279.359	-232.125, -278.284	-222.045, -266.897
2ClBSA	-172.322, -223.214	-222.86, -271.451	-221.671, -270.723	-213.94, -263.492
4ClBSA	-162.49, -229.227	-204.554, -280.837	-203.953, -280.107	-197.774, -272.292
2BrBSA	-172.012, -223.592	-228.469, -282.67	-227.09, -281.268	-214.711, -265.272
4BrBSA	-162.772, -229.021	-204.264, -281.333	-203.626, -280.591	-197.221, -272.946
2ABSA	-182.857, -224.135	-244.339, -278.208	-243.335, -277.289	-233.439, -269.164
SNA	-199.974, -236.632	-269.138, -295.556	-267.81, -294.541	-257.213, -285.933
SMBA	-170.789, -293.551	-224.97, -349.239	-224.155, -348.356	-216.016, -340.781
Coformer				
Lactam/syn-amide				
2PY	-218.848, -195.349	-283.399, -235.02	-282.361, -234.444	-273.377, -228.659
NMePY	-222.485, -94.4617	-287.427, -130.077	-286.328, -129.441	-276.992, -123.688
VLM	-225.43, -176.87	-291.41, -211.81	-290.31, -211.30	-280.984, -205.703
CPR	-224.175, -178.749	-288.683, -211.988	-287.62, -211.547	-278.301, -206.610
AZL	-225.684, -169.133	-291.766, -200.701	-290.760, -200.260	-281.307, -195.271
NMeCPR	-225.291, -91.2207	-293.083, -126.510	-291.988, -125.872	-282.269, -119.762
2HP	-221.826, -234.175	-291.149, -285.17	-291.200, -285.163	-280.897, -277.665
MeHP	-217.892, -227.594	-287.264, -275.934	-286.07, -275.198	-275.723, -268.169
OMeHP	-230.326, -200.407	-305.811, -223.092	-304.467, -222.800	-292.978, -220.097
N-oxides				
Py-oxide	-202.200, -133.160	-275.530, -173.830	-274.160, -172.940	-263.210, -166.800
INA-oxide	-180.989, -264.513	-248.482, -368.555	-247.186, -366.616	-236.841, -351.385
NAM-oxide	-186.673, -257.840	-248.276, -333.978	-247.135, -332.502	-238.242, -321.215
Pyridine carboxamides				

PAM	-239.812, -230.451	-354.678, -320.601	-352.695, -318.947	-334.594, -304.436
NAM	-190.997, -248.609	-241.210, -317.000	-240.580, -315.590	-243.558, -325.721
INA	-181.132, -252.989	-234.843, -339.711	-233.882, -338.03	-226.108, -325.090
CYT	-257.097, -252.969	-353.516, -336.000	-351.627, -334.486	-337.173, -321.716
Amides				
Succinamide	-160.923, -241.130	-195.284, -273.644	-194.743, -273.230	-190.260, -269.184
Malanomide	-203.242, -264.010	-254.074, -331.425	-253.321, -330.305	-246.485, -320.653
Saccharin	-147.536, -269.902	-181.130, -313.545	-180.603, -312.983	-175.556, -307.571
Urea	-211.196, -224.404	-280.719, -305.093	-279.546, -303.676	-269.024, -290.924
Isoniazid	-204.515, -231.339	-281.88, -304.176	-280.372, -302.718	-269.104, -291.071
Pyrazinamide	-216.332, -248.060	-305.861, -337.249	-304.150, -335.490	-290.678, -320.299
Benzamide	-201.241, -228.280	-264.826, -303.390	-263.622, -301.938	-254.394, -291.108
DMSO	-233.437, -143.003	-304.495, -205.536	-303.314, -204.403	-293.336, -194.880
NN-Dimethyl acetamide	-220.179, -113.021	-285.157, -151.663	-284.081, -150.943	-274.347, -145.121
Tri phenyl phosphine Oxide	-259.870, -121.315	-334.280, -188.836	-333.061, -187.748	-322.491, -177.221
Acid coformers				
BA	-163.486, -259.511	-200.798, -303.011	-200.197, -302.237	-194.896, -296.150
PABA	-184.949, -234.693	-235.158, -279.374	-234.297, -278.353	-226.37, -269.255
POHBA	-168.829, -296.294	-207.394, -365.022	-206.762, -363.880	-201.205, -354.800
Oxalic acid	-149.224, -307.900	-176.319, -373.677	-175.837, -372.804	-172.537, -364.657
Melonic Acid	-154.38, - 284.829	-215.735, -335.212	-214.102-, 334.34	-202.640, -327.206

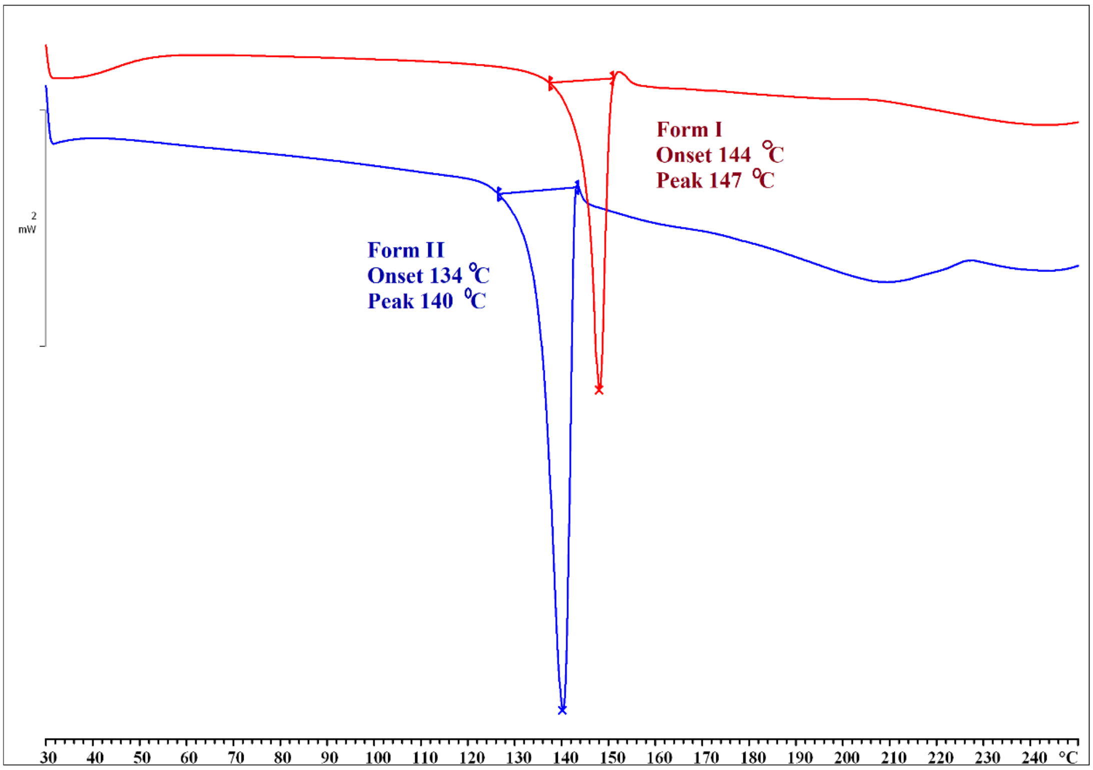

Figure S1 DSC of Form I and Form II of HCT-HP cocrystal polymorphs. Melting endotherm shows that they are monotonically related.

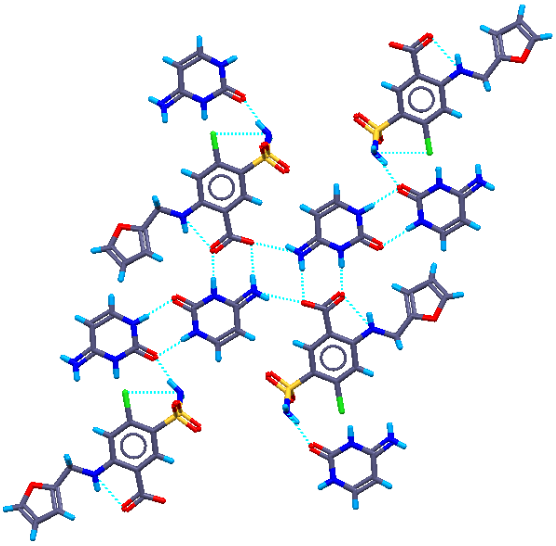

Figure S2 FUROS-CYT cocrystal structure packing, sulfonamide-syn amide and acid-pyridine synthons.

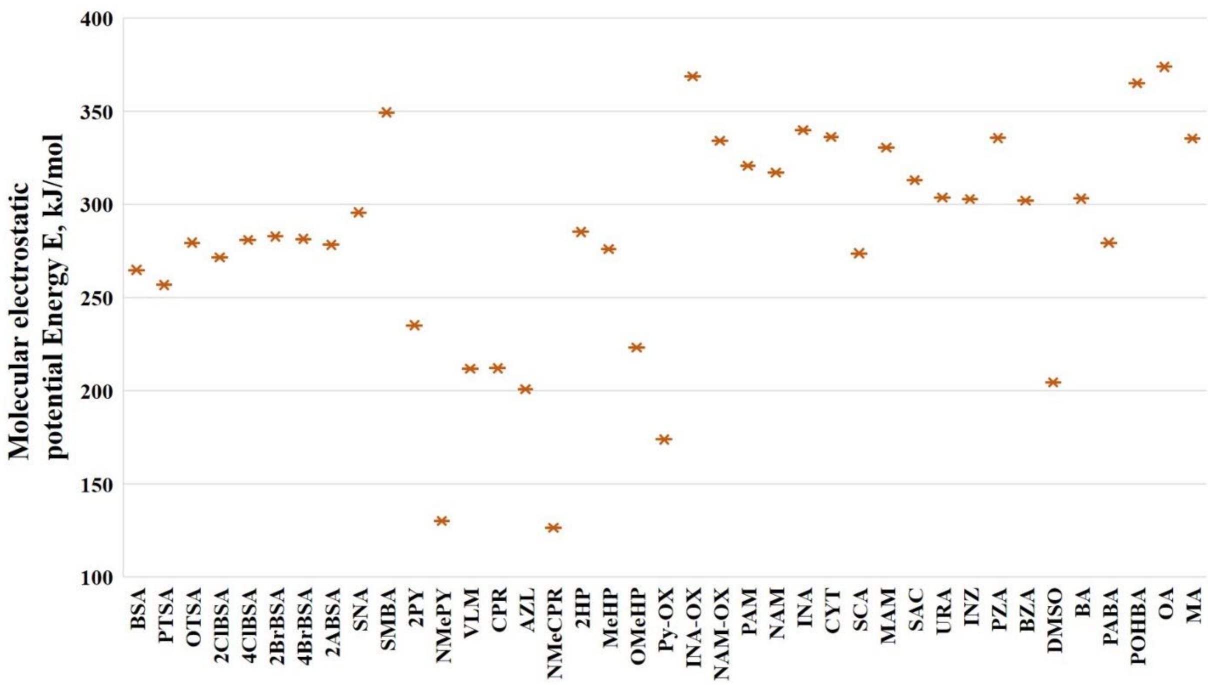
Molecular electrostatic potential Energy E, kJ/mol
400
350
300
250
200
150
100
BSA
PTSA
OTSA
2ClBSA
4ClBSA
2BrBSA
4BrBSA
2ABSA
SNA
SMBA
2PY
NMePY
VLM
CPR
AZL
NMeCPR
2HP
MeHP
OMeHP
Py-OX
INA-OX
NAM-OX
PAM
NAM
INA
CYT
SCA
MAM
SAC
URA
INZ
PZA
BZA
DMSO
BA
PABA
POHBA
OA
MA

(a)

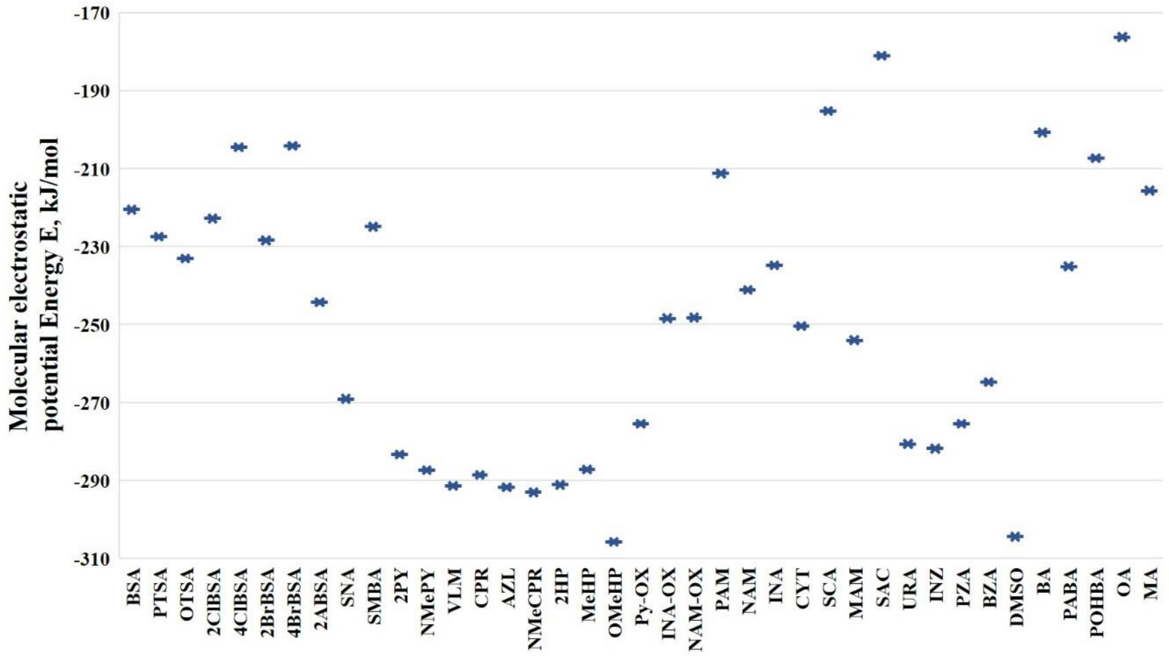
Molecular electrostatic potential Energy E, kJ/mol
-170
-190
-210
-230
-250
-270
-290
-310
BSA
PTSA
OTSA
2ClBSA
4ClBSA
2BrBSA
4BrBSA
2ABSA
SNA
SMBA
2PY
NMePY
VLM
CPR
AZL
NMeCPR
2HP
MeHP
OMeHP
Py-OX
INA-OX
NAM-OX
PAM
NAM
INA
CYT
SCA
MAM
SAC
URA
INZ
PZA
BZA
DMSO
BA
PABA
POHBA
OA
MA

(b)

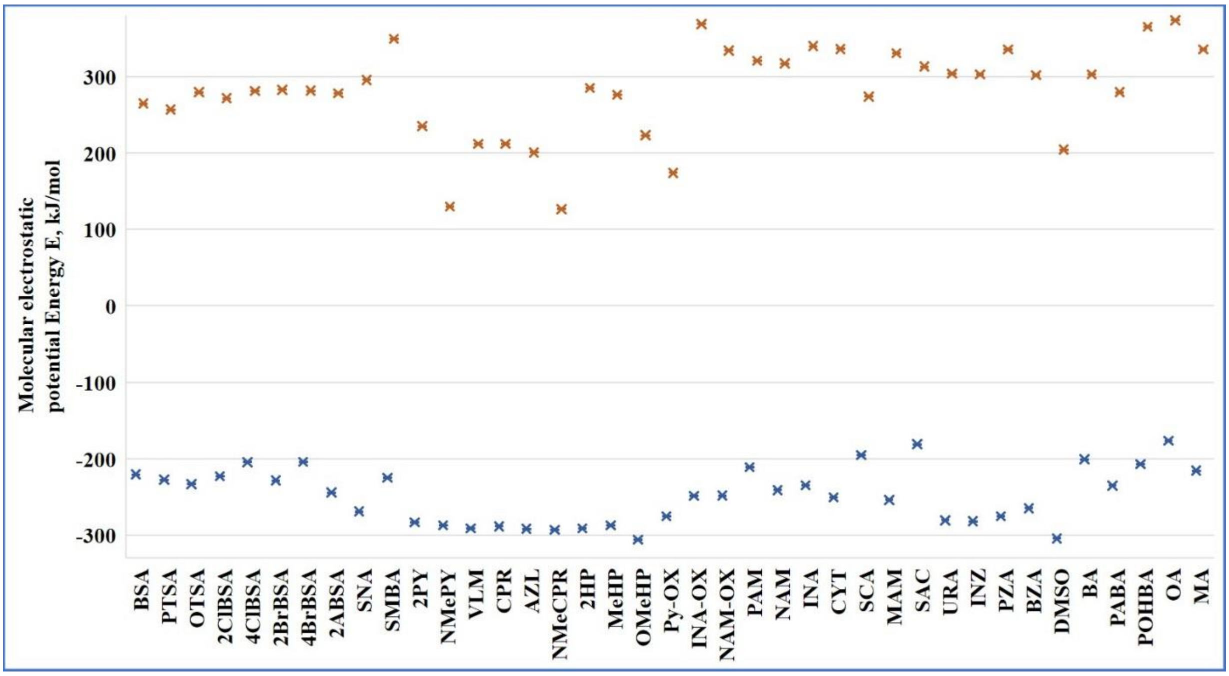

(c)

Figure S3 (a) Negative electrostatic potential (in kJ/mol) of all the donors and acceptors in the present study. These calculations show that lactam and syn-amide is more electronegative that N-oxide coformers. (b) Lactam and syn-amide are less electropositive (in kJ/mol) compared to other coformers. (c) Comparision of the positive and negative electrostatic potential energy.
